# Antineoplastic treatment effect on bone mineral density in Mexican breast cancer patients

**DOI:** 10.1186/s12885-016-2905-x

**Published:** 2016-11-08

**Authors:** Karina Monroy-Cisneros, Julián Esparza-Romero, Mauro E. Valencia, Alfonso G. Guevara-Torres, Rosa O. Méndez-Estrada, Iván Anduro-Corona, Humberto Astiazarán-García

**Affiliations:** 1 Centro de Investigación en Alimentación y Desarrollo (CIAD), Coordinación de Nutrición, Carretera a La Victoria km 0.6, C.P. 83304 Hermosillo, Sonora Mexico; 2 Departamento de Ciencias Químico-Biológicas, Coordinación de Ciencias Nutricionales, Universidad de Sonora, Blvd. Luis Encinas y Rosales S/N, Hermosillo, Sonora Mexico; 3 Centro Estatal de Oncología (CEO), Reforma final y Paseo Río San Miguel, C.P. 83280 Hermosillo, Sonora Mexico

**Keywords:** Breast neoplasm, Bone resorption, Menopause, Calcium, Chemotherapy

## Abstract

**Background:**

Breast cancer is the most deadly malignancy in Mexican women. Although treatment has improved, it may significantly affect bone mineral status in those who receive it. The aim of this study was to assess the impact of cancer treatment on bone mineral density (BMD) and bone mineral content (BMC), in patients with breast cancer and explore the interaction of menopausal status and clinical stage with cancer treatment on such changes.

**Methods:**

A quasi-experimental design was applied with measurements before and after a chemotherapy treatment in 40 patients with primary diagnosis of invasive breast cancer. BMD and body composition measurements were taken by dual X-ray absorptiometry (DXA) and changes in these variables due to therapy were analyzed using mixed regression for repeated measurements.

**Results:**

Significant loss was found in femoral neck and L2-L4 BMD (*p* < 0.001). Patients diagnosed with osteopenia or osteoporosis received calcium + vitamin D supplementation (600 mg/200 IU day). It showed a protective effect in the decrease of femoral neck BMD and total BMC. BMD loss in both femoral neck and L2-L4 BMD was higher in premenopausal women: 0.023 g/cm^2^ in femoral neck and 0.063 g/cm^2^ in L2-L4 (*p* < 0.001), while in postmenopausal women BMD loss was 0.015 g/cm^2^ in femoral neck and 0.035 g/cm^2^ in L2-L4 (*p* = 0.021 and *p* = 0.001 respectively). Change in lumbar spine BMD was prominent in premenopausal women with advanced clinical stage (IIB, IIIA, IIIB): 0.066 g/cm^2^ (*p* = 0.003).

**Conclusion:**

The antineoplastic breast cancer treatment with chemotherapy had a negative impact on BMD, in premenopausal women overall, although a differential effect was found according to clinical stage and calcium supplementation status.

## Background

Breast cancer is considered the neoplasm with the highest incidence and mortality rate in women worldwide. One million seven hundred thousand new cases were recorded in 2012, whereby is the second most common cancer overall and ranks fifth cause of death (522000 deaths) [1].

Although the incidence of breast cancer in Latin America is lower than those in the United States and the European Union, the mortality proportion regarding in those with the disease is high (>30 %), while in Mexico is almost 40 %. Disparities in results are mainly explained by the high prevalence of women diagnosed in advanced stages. For example, 60 % of breast cancer diagnoses are in early stages in the United States and 10 % in Mexico. This explains the most adverse side-effects of the disease and anticancer treatment in those with more advanced stages [2, 3].

During breast cancer, bone tissue is one of the most affected, mainly by ovarian failure, secondary to chemotherapy. The role of estrogens in bone metabolism is important as they stimulate bone formation by promoting osteoprotegerin production (bone formation induction factor), regulating the synthesis of active cytokines in the skeleton (promoted in part by the neoplasm itself) and by apoptosis inhibition of osteoblasts and osteocytes. Thus, estrogen deficiency reduces osteoprotegerin production which causes uncontrolled activity of osteoclasts increasing bone resorption [4, 5].

Some studies have reported differential effects in treatment response and survival, due to different body weight and its distribution by ethnicity [6, 7]. Given the fact that the Mexican population is genetically diverse, along with the knowledge that environmental and demographic factors may also affect susceptibility to treatment, the objectives of this study was to evaluate the impact of initial anticancer therapy on bone mineral status in Mexican women, by estimating the change in BMD in the femoral neck, lumbar spine (L2-L4) and total BMC. An additional objective was to evaluate the differential effect due to the clinical stage and menopausal status on BMC and BMD.

## Methods

The design is based on a quasi-experimental prospective follow-up study of women with primary diagnosis of invasive breast cancer who attended at the State Center of Oncology (CEO from the acronym in Spanish) in Hermosillo, Sonora, to begin the anticancer treatment. The initial six-months of chemotherapy treatment was assay. Women were invited if they showed no metastasis or other diseases which affected their body composition such as hypo and hyperthyroidism, fractures and/or disabling injuries or those who had received chemotherapy in the past. Patients were also excluded from the analysis if they developed metastasis during the study course and/or discontinued their treatment.

The protocol was approved by the Research Ethics Committee of the CEO and Ethics Committee of the Research Center for Food and Development (CIAD from the acronym in Spanish). The recruited volunteers were requested to sign an informed consent form. Information on clinical stage, treatment schedule, menopausal status (menstruation absence for over a year before treatment), socioeconomic status (determined by the social work department of the hospital in terms of monthly income, housing characteristics and access to basic services, etc.) and demographic data were taken from the clinical records of the participants at CEO with their previous permission. Anthropometric, body composition and BMD measurements were analyzed at CIAD facilities.

Body composition and BMD were determined using a dual X-ray absorptiometry (DXA-MD + Lunar Co., Madison, WI). Precision of that measures was Coefficient Variation (CV) <2 %. Three measurements (scans) were obtained, one for the whole body to assess the body composition and two in the lumbar spine (L2-L4) and femoral neck to determine the BMD. The results of body composition were reported in kilograms and BMD in g/cm^2^. All measurements were performed by trained and certificated technical staff before and after 6 months of receiving antineoplastic therapy. Women with diagnosed osteopenia or osteoporosis at baseline (*n* = 10) received a prescription of calcium and vitamin D_3_ supplementation (600 mg/200 IU day).

A total of 57 women were recruited from August 2007 to January 2008 (24 women) and from February to August 2009 (33 women). Three women dropped out the treatment and 14 more were excluded from the study due to metastases development during the tracking period; therefore, 40 patients were included in analyses.

### Statistical analysis

All analyzes were performed with NCSS software version 7.03 (2007). A paired t-test was applied to analyze the changes of the study parameters before and after treatment. Normal distribution of the quantitative variables was evaluated using histograms; all response variables exhibit Gaussian distribution. To evaluate the treatment related changes of total BMC, BMD on femoral neck and L2-L4, separate mixed regression models for repeated measures were used. Interaction terms of hypothesized variable (treatment) with menopausal status (pre or post), calcium supplementation (yes or no) and clinical stage phase were considered at *p* ≤ 0.05. When a significant interaction was found, stratified analyses were conducted. The clinical stage was analyzed as a dichotomous variable due to the small number of subjects in some stages. Stages I and IIA in one category and stages IIB, IIIA and IIIB in a second one. Statistical significance was considered at *p* ≤ 0.05.

## Results

The patients age distribution was 32 to 61 years; 19 were premenopausal and 21 postmenopausal. The average age of menarche was 12 years. Five patients were nulliparous, 28 reported using oral contraceptives (>3 years), 6 recognized to be regular smokers (daily) at the diagnostic time and 7 accepted had consumed alcohol occasionally (>1 time/week). Regarding education status, 11 women were enrolled beyond the basic level (high school or technical degree). Regarding socioeconomic status, 62.5 % were in low and 37.5 % in medium or high.

Five women were found in the stage I, 12 in IIA, 8 in IIB, 9 in IIIA and 6 in IIIB (Classification TNM) [8]. All patients received chemotherapy. Prescribed schedules were FAC (5-Fluorouracil, Adriamycin, Cyclophosphamide), FEC (5-Fluorouracil, Epirubicin, Cyclophosphamide) or DAC (Docetaxel, Adriamycin, Cyclophosphamide). Of the premenopausal patients 68 % become amenorrheic. Radiotherapy was additionally administered to 25 patients and 7 were eligible for monoclonal antibodies treatment (Trastuzumab); one of them not receive it for cardiac issues. The clinical pathological characteristics and chemotherapy schemes distribution describes at Table 1.

**Table 1:** Patient characteristics and clinicopathologic features. (*n* = 40)

	Premenopausal (*n* = 19)	Postmenopausal (*n* = 21)
Age (mean)	43	53.55
Basal BMI (kg/m^2^, mean)	28.04	31.68
Clinical stage		
I	3	2
IIA	5	7
IIB	3	5
IIIA	5	4
IIIB	3	3
Histological subtype		
Invasive ductal carcinoma	18	19
Invasive lobulillar carcinoma	1	2
Treatment		
Neoadyuvant	9	9
Adyuvant	10	12
Molecular subtypes (*n* = 28)^a^		
Luminal A (RE+, RP+/-, HER2neu-)	1	3
Luminal B (RE+, RP+/-, HER2neu+)	4	5
HER2 (RE-, RP+/-, HER2+)	5	2
Triple negative (RE-, RP-, HER2-)	5	3
Chemotherapy scheme^b^		
-FAC (5-Fluorouracile, Adriamicin, Cyclophosphamide)	15	17
-FEC (5-Fluorouracile, Epirrubicin, Cyclophosphamide)	-	1
-FAC + P(5-Fluorouracile, Adriamicin, Cyclophosphamide + Paclitaxel)	3	1
-FA (5-Fluorouracile, Cyclophosphamide)	1	-
-DAC (Docetaxel, Adriamicin, Cyclophosphamide)	-	2

Data in table are number of cases

^a^Because patient recruitment started in 2007 and immunohistochemistry determination of hormone receptors was instituted as part of the routine battery of tests in 2008, not all underwent such a test

^b^National Comprehensive Cancer Network (NCCN) Clinical guidelines

Table 2 displays t-score of BMD distribution on premenopausal and postmenopausal patients before and after to receive antineoplastic treatment at femoral neck and lumbar spine (L2-L4).

**Table 2:** Bone Mineral Density (BMD) T-score^a^ distribution and change at six months of antineoplastic breast cancer treatment. (*n* = 40)

Menopausal status	Bone region	Normal (T score > -1)	Osteopenia (T score < -1 > 2.5)	Osteoporosis (Tscore < -2.5)
Premenopausal (*n* = 19)	Femoral neck			
	Basal	12	7	-
	6 months treatment	17	2	-
	Lumbar spine (L2-L4)			
	Basal	9	7	3
	6 months treatment	4	10	5
Postmenopausal (*n* = 21)	Femoral neck			
	Basal	21	-	-
	6 months treatment	16	5	-
	Lumbar spine (L2-L4)			
	Basal	21	-	-
	6 months treatment	21	-	-

Data in table are number of cases

^a^WHO cut-off points. T-score is defined as BMD Standard Deviations (SD) respect to 20–39 years old health population of same gender

Table 3 shows parameters from the unadjusted analysis. Significant loss was observed in femoral neck and L2-L4 BMD (*p* < 0.001). In BMC the change did not reach statistical significance (*p* = 0.066).

**Table 3:** Characterization of BMD and BMC parameters in breast cancer patients (*n* = 40)

Parameter	Basal^a^ (No treatment)	6 months^a^ (After treatment)	Δ^b^	*p* ^c^	Confidence Interval 95 %
BMD femoral neck (g/cm^2^)	1.02 ± 0.1	1.001 ± 0.1	−0.018	<0.001	(−0.026, −0.011)
BMD L2-L4 (g/cm^2^)	1.19 ± 0.2	1.15 ± 0.1	−0.048	<0.001	(−0.063, −0.033)
BMC (kg)	2.46 ± 0.34	2.43 ± 0.34	−0.032	0.066	(−0.065, 0.002)

*BMD*Bone Mineral Density, *BMC* Bone Mineral Content

^a^Mean ± SD

^b^Δ = measurement 2- measurement 1

^c^paired t test

A protective effect in the reduction of BMD at femoral neck and total BMC due calcium supplementation was found. In women supplemented there was no significant decrease in femoral neck BMD or total BMC; but in L2-L4 BMD which was significant (*p* = 0.003) despite calcium + vitamin D supplementation. Conversely, women who did not receive calcium supplement, showed significantly loss in femoral neck BMD, L2-L4 BMD and total BMC (*p* < 0.001, *p* < 0.001 and *p* = 0.039) (Table 4).

**Table 4:** Antineoplastic treatment effect on BMD and BMC based on oral calcium and vitamin D_3_ supplementation in breast cancer patients (*n* = 40)

Parameter	No calcium supplement (*n* = 30)			Calcium supplement (*n* = 10)^b^		
	β^a^	*p*	Confidence Interval 95 %	β^a^	*p*	Confidence Interval 95 %
BMD femoral neck (g/cm^2^)	−0.03	<0.001	(−0.036, −0.024)	0.006	0.344	(−0.007, 0.018)
BMD L2-L4 (g/cm^2^)	−0.055	<0.001	(−0.076, −0.035)	−0.033	0.003	(−0.052, −0.013)
BMC (kg)	−0.047	0.039	(−0.092, −0.003)	<0.001	0.987	(−0,052, 0.052)

*BMD* Bone Mineral Density, *BMC* Bone Mineral Content

^a^Determined by mixed model regression for repeated measures. Calcium supplement and treatment interaction (*p* ≤ 0.05)

^b^Calcium and vitamin D_3_ supplementation (600 mg/200 IU day)

There was also interaction between treatment effect vs. menopausal status and vs. clinical stage (*p* < 0.05). Consequently, all the analyses were completed in the stratus of the interaction variables (Table 5). BMD loss was significant in premenopausal and postmenopausal women: −0.023 g/cm^2^ in femoral neck and −0.063 g/cm^2^ in L2-L4 (*p* < 0.001) in premenopausal women and −0.015 g/cm^2^ in femoral neck (*p* = 0.021) and −0.035 g/cm^2^ in L2-L4 (*p* = 0.001) in postmenopausal. In premenopausal patients decrease in L2-L4 BMD was directly related with clinical stage: −0.059 g/cm^2^ on stage I, IIA (*p* = 0.006) and −0.066 g/cm^2^ on stage IIB, IIIA, IIIB (*p* = 0.003). However, in femoral neck, a reverse pattern was observed, where BMD was −0.030 g/cm^2^ on stage I, IIA (*p* = 0.004) and −0.017 g/cm^2^ on stage IIB, IIIA, IIIB (*p* = 0.007). Also this trend remained between postmenopausal women, with significant changes in stages I, IIA (*p* = 0.037 for femoral neck and *p* < 0.001 for L2-L4).

**Table 5:** Antineoplastic treatment effect on BMD and BMC depending on menopausal status and clinical stage in breast cancer patients (*n* = 40)

Parameter		Premenopause (*n* = 19)			Postmenopause (*n* = 21)		
		β^a^	*p*	Confidence Interval 95 %	β^a^	*p*	Confidence Interval 95 %
BMD femoral neck (g/cm^2^)^b^	All	−0.023	<0.001	(−0.032, −0.013)	−0.015	0.021	(−0.027, −0.002)
Clinical stage I, IIA (*n* = 17)^c^		−0.030	0.004	(−0.047, −0.013)	−0.018	0.037	(−0.034, −0.001)
Clinical stage IIB, IIIA, IIIB (*n* = 23)^c^		−0.017	0.007	(−0.029, −0.006)	−0.0123	0.198	(−0.032, 0.008)
BMD L2-L4 (g/cm^2^)^b^	All	−0.063	<0.001	(−0.086, −0.039)	−0.035	0.001	(−0.054, −0.016)
Clinical stage I, IIA (*n* = 17)^c^		−0.059	0.006	(−0.095, −0.023)	−0.043	<0.001	(−0.059, −0.026)
Clinical stage IIB, IIIA, IIIB (*n* = 23)^c^		−0.066	0.003	(−0.102, −0.029)	−0.029	0.088	(−0.062, 0.005)
BMC (kg)^b^	All	−0.027	0.29	(−0.078, 0.025)	−0.036	0.140	(−0.085, 0.013)
Clinical stage I, IIA (*n* = 17)^c^		−0.019	0.66	(−0.118, 0.079)	−0.005	0.895	(−0.088, 0.078)
Clinical stage IIB, IIIA, IIIB (*n* = 23)^c^		−0.032	0.33	(−0.103, 0.038)	−0.059	0.078	(−0.126, 0.008)

*BMD* Bone Mineral Density, *BMC* Bone Mineral Content

^a^Determined by mixed model regression for repeated measures

^b^Menopausal status and treatment interaction (*p* ≤ 0.05)

^c^Menopausal status and clinical stage interaction (*p* ≤ 0.05)

## Discussion

Estrogen activity is linked to bone metabolism and some chemotherapeutic agents such as cyclophosphamide which is related with bone resorption because of ovarian failure in premenopausal women related with this agent [9, 10]. Although almost all volunteers showed a significant loss in BMD, the impact was greater in premenopausal women. Furthermore, the decrease in BMD is higher than that reported by other authors during the same post-diagnosis period (6 months): For example, Shapiro et al.[9] reported 4 % lumbar spine BMD loss, in the present study it was similar (almost 5 %). Other studies reported comparable decline over a period of two years [11, 12].

In postmenopausal patients, BMD change was −3.08 %. Powles et al. [13] in a similar group of women, found that the change in the lumbar spine BMD was only −0.37 %. Since there is lack of information in the literature about the magnitude of these changes in Mexican population under an internationally standardized chemotherapy regimen [14, 15], we believe that perhaps other causes such as genetic factors could be involved and can explain the differential effect found in this study with respect to others. Other studies have found that body composition differs significantly between Hispanic and Caucasian breast cancer female patients, however, neither group of countries have established guidelines for the administration of antineoplastic drugs based on body composition, in which case toxicity could be different for these groups [16]. Since the effective concentration of these antineoplastic drugs is affected by the amount of lean tissue [17, 18], it is believed that this can be a key element to elucidate the reason for the differences between the magnitude of the changes on BMD found by others researchers and the present study . The body antineoplastic concentration reached could also affect changes in body weight and body composition components such as BMD, fat mass, and fat free mass. However, other factors such as the diet may be involved.

Some patients started treatment with osteopenia, and calcium was part of their treatment. It appears that calcium showed a significant protective effect on the femur, but not on the lumbar spine. The explanation to the lower BMD loss on femoral neck than lumbar spine in both groups of women (calcium supplemented and no-supplemented) may be the same. The proportion of trabecular-cortical tissue and bone turnover rate are different in each skeletal region. The pathophysiology of osteoporosis [19, 20] indicates that about 25 % of trabecular bone (cancellous) is replaced every year, while cortical bone (laminar) is replaced at a level of 3 %. This is the reason why bone resorption degree is calculated using BMD as a reference at the proximal femur (neck, trochanter) and spine. Both sites contain high proportion of cancellous tissue. Although these points have a high content of trabecular bone compared to the rest of the skeletal system, there is an important difference in their composition. The fact that proximal femur has 30–50 % of trabecular tissue whereas the lumbar spine has 85 % explains why the lumbar spine is more susceptible to the effect due to decreased estrogen level, so that bone tissue resorption at L2-L4 resulted exacerbated. There is a greater release of calcium into the bloodstream, which can maintain parathyroid hormone (PTH) levels marginally low; preventing activation and mobilization of enough vitamin D on lumbar spine tissue to stimulate the calcium bond. On the other hand, the femoral neck bone turnover rate is lower and calcium supplied along with activation and mobilization of vitamin D with that level of PTH, is enough to raise the rate of mineralization at the newly remodeled tissue.

A recent finding indicates that the involvement of estrogens and its protective effect on trabecular tissue metabolism is much higher than that in cortical tissue, which could explain the higher susceptibility of cancellous tissue to ovarian ablation, regardless of differences in the rate of bone turnover between these tissues [21]. In addition to primary chemotherapeutic treatment scheme, which contributes to bone resorption, breast cancer patients received anti-estrogen therapy for a five-year period. These drugs also favored the decrease in BMD. Confavreux et al. [22] found that the patients who underwent treatment with anastrozole had a decrease of 3.3 % in lumbar spine BMD and 2.8 % in femoral neck in a one year period. Some investigations have published similar effects as those of Letrozole, Tamoxifen and Exemestane treatments [23–25].

Another consideration in decreasing BMD is the increase in body weight in patients. As previously reported [26], this patients group decreased lean mass, while premenopausal women increased body weight and adipose tissue. Since one of the mechanisms that stimulates the bone remodeling is the body calcium demand, it appears that the higher increase of body mass the greater the rate of calcium mobilization from bone matrix, combined with poor bone remodeling by estrogen loss which can help control osteoclastic activity [20]. However, the fact that fat tissue showed higher increase than lean tissue, could result in increased toxicity due to antineoplastic drugs, because they can have a greater effect since the dosage thereof is calculated according to total body weight but its real concentration in the body is affected for the lean tissue proportion. This could be a reason to suggest the evaluation of sarcopenia status in patients with cancer as well as chemotherapy dose adjustments [17, 18].

An additional finding is the clinical stage effect on BMD. In the initial chemotherapy scheme there are no important differences in the antineoplastic doses and their combinations between clinical stages that could result in a different effect on estrogen concentration in the 6-month-follow-up period. Variations in treatment are prescribed depending on factors such as the type of carcinoma (ductal, lobular, etc.), molecular subtype and the age of the patients [15]. Patients in medium (IIB) or advanced clinical stages (IIIA, IIIB) are the most affected in regards to BMD, which could be explained by the tumor’s increased production of catabolic cytokines as well as by the body’s induced response [27, 28]. There is also the effect mediated by IL-1, IL-6, IFNγ and TNFα, that increases the invasiveness of the tumor on bone tissue. These cytokines also contribute to the loss of lean tissue in cancer patients with cachexia [29]. The loss of lean tissue can be masked by increased body fat in breast cancer patients [30].

The evaluation of BMD is not a routinely part of the treatment in cancer patients, even considering the harmful effects of anticancer and anti-estrogen on bone matrix. Breast cancer women may benefit from a proper diagnosis, plus the fact that they could also be given antirresorptive drugs with no additional cost to the patient that is already covered by the public government health programs. One might speculate that loss in BMD is not so relevant when considering the cost-benefit ratio of cancer treatment, in short term. In our study we found 4.97 % of BMD loss at the lumbar spine in six months. Saarto et al. [12] evaluated a group of patients and after two years found a similar decrease of BMD; after ten years, 23 % of them developed osteoporosis [31].

Health institutions worldwide should implement the evaluation of BMD in patients with breast cancer as part of the treatment regimen as suggested by the American Society of Clinical Oncology (ASCO) [32]. In addition, antiresorptive therapies could prevent bone metastasis [33]. Due to the alert issued in 2011 by the Food and Drug Administration (FDA) regarding the kidney damage in some cases, the ASCO recommended the limited use of bisphosphonates to the patients with bone metastases, restricting their prescription in patients with osteopenia or osteoporosis with no evidence of bone destruction diagnosed by X-ray Computed Tomography (CT) or Magnetic Resonance Imaging (MRI) [34]. However, since the high loss in BMD found in this study, it is suggested that the treatment could be implemented in patients with osteopenia and osteoporosis at baseline; while monitoring renal function, as routinely done for patients receiving cisplatin in chemotherapy scheme [35]. This strategy may reduce long-term costs, would impact positively on the quality of life and survival of these women.

A few limitations should be mentioned. Due to the nature of the study design and the fact that the chemotherapy treatment scheme was standardized, it is not possible isolate completely the effect thereof on the measured parameters considered. For this reason, it is unknown the influence of the tumor on BMD changes and its induced catabolic body response. Furthermore, the protective effect of calcium supplementation on demineralization in the femoral neck must be interpreted with caution since supplemented patients who initiated treatment had osteopenia. It is considered that women who begin the chemotherapy scheme with normal BMD calcium supplementation could not have the same effect. One more limitation was the small sample size in this study which confines its generalizability. Therefore, results need to be replicated in larger studies.

## Conclusions

This study showed evidence of a significant reduction of BMD in both femoral neck and L2-L4. Due to treatment and menopausal status interaction, bone resorption was higher in premenopausal patients. Also, calcium supplementation exerted a protective effect by reducing the loss of total BMC and femoral neck BMD. The treatment and clinical stage interaction on L2-L4 BMD loss suggested that the bone resorption could be due not only to the antineoplastic drugs, but also to the disease itself, which could have had an effect even before clinical signs such as the bone metastasis and cachexia appeared. However, further studies focused on tumor response at the initial phase of treatment are required to elucidate the extent of malignancy *per se*.

## Abbreviations

BMC: Bone mineral content
BMD: Bone mineral density
CEO: State Oncology Center
CIAD: Food and Development Research Center
DXA: Dual X Absorptiometry
